# Endothelin-1 Enriched Tumor Phenotype Predicts Breast Cancer Recurrence

**DOI:** 10.1155/2013/385398

**Published:** 2013-06-12

**Authors:** Deimante Tamkus, Alla Sikorskii, Kathleen A. Gallo, David A. Wiese, Cheryl Leece, Burra V. Madhukar, Simona C. Chivu, Shalini Chitneni, Nikolay V. Dimitrov

**Affiliations:** ^1^Department of Medicine, Michigan State University, B409 Clinical Center, East Lansing, MI 48824-1317, USA; ^2^Department of Statistics and Probability, Michigan State University, 432A Wells Hall, East Lansing, MI 48824-1317, USA; ^3^Department of Physiology, Michigan State University, 4164 Biomedical Physical Sciences, East Lansing, MI 48824, USA; ^4^Department of Pathology, McLaren Regional Medical Center, 3490 Calkins Road, Flint, MI 48532, USA; ^5^Michigan State University, B234 Life Science Building, East Lansing, MI 48824-1317, USA; ^6^Pediatrics and Human Development, Michigan State University, B238 Life Science Building, East Lansing, MI 48824-1317, USA; ^7^Michigan State University, 401 W Greenlawn Avenue, Lansing, MI 48910, USA; ^8^Department of Medicine, Michigan State University, B413 Clinical Center, East Lansing, MI 48824-1317, USA

## Abstract

*Introduction*. Breast cancer recurrence can develop years after primary treatment. Crosstalk between breast cancer cells and their stromal microenvironment may influence tumor progression. Our primary study aim was to determine whether endothelin-1 (ET-1) expression in tumor and stroma predicts breast cancer relapse. The secondary aim was to determine ET-1/endothelin receptor A (ETAR) role on signaling pathways and apoptosis in breast cancer. *Experimental Design*. Patients with histologically documented stages I–III invasive breast cancer were included in the study. ET-1 expression by immunohistochemistry (IHC) in tumor cells and stroma was analyzed. Association between ET-1 expression and clinical outcome was assessed using multivariate Cox proportional hazard model. Kaplan-Meier curves were used to estimate disease-free survival (DFS). In addition, the effect of ET-1/ETAR on signaling pathways and apoptosis was evaluated in MCF-7 and MDA-MB-231 breast cancer cells. *Results*. With a median followup of 7 years, ET-1 non-enriched tumor phenotype had a significant association with favorable disease-free survival (HR = 0.16; 95% CI 0.03–0.77; *P* value <0.02). ER negativity, advanced stage of disease and ET-1-enriched tumor phenotype were all associated with a higher risk for recurrence. Experimental study demonstrated that ET-1 stimulation promoted Akt activation in MCF-7 and MDA-MB-231 cells. Furthermore, silencing of ETAR induced apoptosis in both hormone receptor negative and hormone receptor positive breast cancer cells. *Conclusions*. We found ET-1 expression in tumor and stroma to be an independent prognostic marker for breast cancer recurrence. Prospective studies are warranted to examine whether ET-1 expression in tumor/stroma could assist in stratifying patients with hormone receptor positive breast cancer for adjuvant therapy.

## 1. Introduction

Breast cancer metastases can develop years after primary tumor treatment [1]. Current adjuvant systemic or regional therapies eliminate a majority of cancer cells. However, a subset of cancer cells, which are not effectively eradicated by the treatment, may maintain their potential for further growth. Tumor cells may interact with their stromal microenvironment to either facilitate or delay tumor dissemination, thus influencing tumor recurrence in breast cancer patients [2]. Thirty to 40% of patients with early-stage breast cancer at the time of diagnosis have disseminated tumor cells detected in the bone marrow [3]. The majority of these disseminated tumor cells die, but some of them remain dormant and have the capacity to manifest as clinical tumor recurrences. In order to survive the dissemination process and avoid apoptotic clearance, tumor cells would be expected to have robust signaling through prosurvival pathway [4]. 

Endothelins (ETs) are a family of small peptides whose involvement in tumor growth by modulation of proliferation, apoptosis, angiogenesis and invasion has been documented [5]. In addition to tumor cells, the surrounding stromal cells, including macrophages and endothelial cells, may also express ETs and their receptors (ETAR and ETBR) [6, 7]. ET-1 has been shown to foster tumor-stroma interactions and promote the malignant tumor microenvironment [8–12]. In addition, ETs have been implicated in resistance to cancer therapy and tumor recurrence [13, 14]. The exact biologic mechanism by which ETs expressing tumor cells escape therapeutic agents in breast cancer is not well defined. 

The primary goal of our study was to evaluate ET-1 expression in breast cancer cells and surrounding stroma and determine its effect on recurrence risk. A secondary aim was to determine ET-1/ETAR signaling pathway effect on apoptosis in breast cancer cells. 

## 2. Methods

### 2.1. Study Population

We conducted a retrospective, multicenter study. Patients with histologically documented stages I–III invasive breast cancer were identified in the clinic and electronic medical records from three medical centers affiliated with Michigan State University. The study was approved by local institutional review board (IRB). Informed consent was obtained from patients. Total study population consisted of 91 patients. The date of primary diagnosis ranged from August 1994 until March 2009. Demographics, clinicopathologic data, treatment information, and recurrence free interval in months were all obtained from medical records and available for statistical analysis. 

### 2.2. Tissue Immunohistochemistry

**Figure 1:**
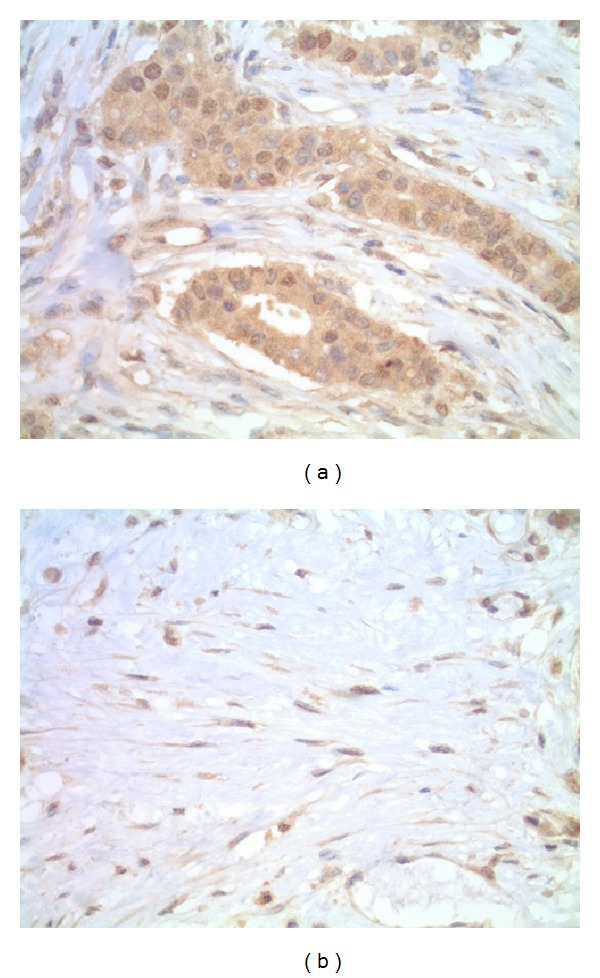
Representative examples of ET-1-enriched tumor phenotype. 3+ IHC staining for ET-1 in tumor cells (a), 2+ IHC staining for ET-1 in stromal cells (b).

Formalin-fixed and paraffin-embedded tumor samples of the patients were cut at 4 *μ*m and prepared by heat induced epitope retrieval (Cell Marque Trilogy, Rocklin, CA) under pressure (Cuisinart model EPC-1200PC). The sections were immunostained for ET-1 by a multistep semiautomatic procedure (Ventana NexES, Tucson, AZ) using ET-1 monoclonal mouse antibody (clone TR.ET.48.5, Thermo Scientific Pierce Antibodies, Rockford, IL) incubated at a 1 : 250 dilution for 30 minutes followed by iVIEW DAB detection (Ventana). A known ET-1 positive prostate cancer tissue was used as a positive control for each run; specificity of the antibody was confirmed by omitting the primary antibody and replacing it with mouse IgG as a negative control. Paired sections were stained routinely with hematoxylin and eosin (H&E) for the histologic confirmation of the primary tumor. The expression of cytoplasmic ET-1 was scored in both the epithelial and adjacent stromal compartments. Staining intensity of 3+ by IHC was scored as positive in tumor; 0, 1+, or 2+ by IHC was scored as negative in tumor. At least 10% of the tumor cells had to have 3+ staining by IHC to be considered as positive. Staining intensity of 2+ or 3+ by IHC was scored as positive in stroma, and 0 or 1+ by IHC was scored as negative in stroma. ET-1-enriched tumor phenotype was defined as (1) ET-1 3+ (strong staining) in tumor or (2) ET-1 score of 3+ (strong staining) or 2+ (moderate staining) in stroma. Representative images of ET-1-enriched tumor phenotype are shown in Figure 1.

### 2.3. Statistical Analysis

Primary endpoint-disease, free survival (DFS), was defined as time from mastectomy or lumpectomy until invasive local, regional or distant recurrence. The distributions of the outcomes of recurrence and time to recurrence as well as potential explanatory variables (ET-1 status, staging, hormone receptor status, and treatment) were summarized and compared for those patients with and without recurrence using *t*-tests, chi-square, or Fisher's exact tests. The comparisons of the outcomes and covariates (explanatory variables listed above except for ET-1 status) were also performed according to ET-1 status. Multivariate Cox proportional hazard model was employed to test for the association of ET-1 status with the probability of recurrence, and time to recurrence. Variables included in the model were ET-1 status, ER/PR status, HER2 status, age, and tumor and nodal stage. The effects of ET-1 were further explored in the ER/PR positive subset of patients using Cox proportional hazard model. 

### 2.4. Cell Lines and Culture

Two human breast cancer cell lines MCF-7 and MDA-MB-231 were obtained from the American Type Culture Collection (ATCC, Rockville, MD). MCF-7 cells were maintained in Dulbecco's modified eagle medium (DMEM) and supplemented with 10% (v/v) fetal bovine serum (FBS), and MDA-MB-231 were maintained in DMEM/F12 medium containing 10% (v/v) FBS. Cells were grown at 37°C at 5% CO_2_ and subcultured every 3 days.

### 2.5. Antibodies and Western Blotting

Cell lysates obtained from cell cultures were subjected to SDS-polyacrylamide gradient gel electrophoresis (7.5–12.5%) and revealed by Western blotting using antibody Akt (1 : 1000) and phosphor-Ser-473-Akt (1 : 500). The membranes were reprobed with anti-*β*-tubulin to assure equal amounts of protein.

### 2.6. Apoptosis Assays

The Annexin V (early apoptotic) and propidium iodide (late apoptotic) double staining method was used. Cells were harvested with trypsin-EDTA and then stained with the FITC Annexin V Apoptosis Kit according to the manufacturer's instructions (BD Biosciences, San Diego, CA). Cells were subjected to flow cytometric analysis with a flow cytometer (Becton Dickinson model FACSCalibur). 

### 2.7. Immunofluorescence

Following culture on Lab-tek chamber slides, cells were fixed with cold methanol and stained with primary antibody p-Akt (1 : 50), secondary antibody (1 : 300) DyLight 549 goat anti-rabbit (Jackson ImmunoResearch, West Grove, PA). Images were taken using an Olympus Fluoview FV1000 confocal laser scanning microscope (MSU Confocal Microscopy facility). 

### 2.8. siRNA against ETAR

Transient knockdown of ETAR was performed using siRNA against ETAR. MCF-7 or MDA-MB-231 cells were transfected with 100 nmol/L small interfering RNA (siRNA) duplexes against ETAR mRNA (Mission siRNA, Sigma, St Louis, MO) or universal negative control siRNA obtained commercially (Sigma). siRNA transfection using N-ter (Sigma) was done according to manufacturer's protocol. Cells were harvested 48 hours later, and ETAR protein levels were determined. 

## 3. Results

### 3.1. ET-1 Expression in Breast Tumor and Stroma and Correlation with Clinical Outcome

Table 1 summarizes the characteristics of the study sample. The study included 91 patients with average age at the diagnosis of 55 years (range 32–85). Median followup of patients at the time of analysis was 85.1 months. A total of 31 patients experienced breast cancer relapse. Of those undergoing relapses, 20 patients developed distant metastasis. As expected, higher nodal involvement and advanced stage were associated with the increased risk of relapse (*P* = 0.02). 

Positive ET-1 cytoplasmic staining in tumor cells was detected in 72.5% of cases, while positive ET-1 expression in adjacent stroma was identified in 65.9% of cases. There was a positive correlation between tumor and stromal ET-1 intensity. 76.4% of ET-1 positive tumors had adjacent stroma which strongly/moderately expressed ET-1, while ET-1 negative tumors were surrounded by ET-1 nonexpressing stroma in 65.2% of cases. In our study population, ET-1-enriched tumor phenotype was observed in 84.4% of breast cancers. No statistically significant association was found between tumor/stromal ET-1 expression and ER, PR, HER2/neu receptor status. It should be noted that HER2/neu overexpressed/amplified tumors were underrepresented in our study sample. The unadjusted comparisons of ET-1-enriched versus non-enriched groups were performed. There was no statistically significant difference in clinical, histopathological parameters (age, tumor grade, tumor stage, nodal stage, clinical stage, adjuvant chemotherapy, and endocrine therapy received) in patients with ET-1-enriched or non-enriched tumor phenotype. 

**Figure 2:**
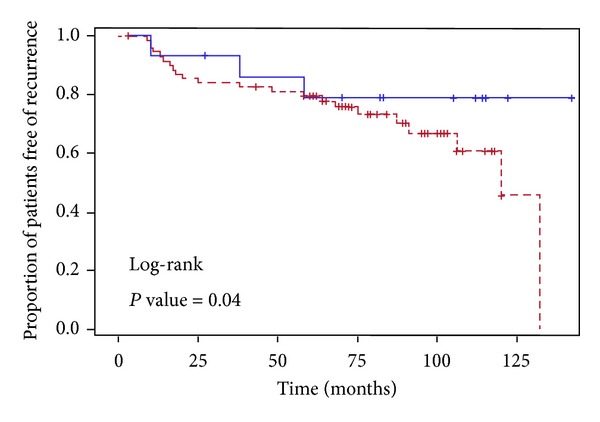
Kaplan-Meier curves for outcomes. Disease-free survival (DFS) in breast cancer patients with ET-1 non-enriched (blue, solid line) and ET-1-enriched tumor phenotype (red, broken line).

DFS was analyzed according to ET-1 expression in tumor and stroma. Patients with ET-1-enriched tumor phenotype showed significantly higher risk for recurrence, while patients with non ET-1 non-enriched tumors had an excellent prognosis (Figure 2). To evaluate the potential interaction between ET-1 expression and disease-free-survival, we tested the significance of interaction in a proportional hazard model adjusted for age, tumor stage, number of positive nodes, ER status and HER 2 status. In the Cox proportional hazard model, ET-1 non-enriched phenotype, ER/PR positivity, and less advanced stage were all associated with lower hazard for recurrence (Table 2). ET-1 non-enriched tumor phenotype had a significant association with favorable disease-free survival (HR = 0.16; 95% CI 0.03–0.77; *P* value <0.02). 

We also conducted the exploratory analysis of a subset of patients who had ER and/or PR positive tumors (*N* = 61). Due to sample size and relatively small cell counts, statistical significance was not reached; however, the directions of the associations found in this subset were consistent with the results obtained in the entire sample. In the ET-1 non-enriched subgroup, 12.5% of patients experienced a recurrence, while for ET-1-enriched cases, 26% had a recurrence. In the Cox model, the hazard ratio for the ET-1 non-enriched phenotype was 0.23, with a wide 95% confidence interval of 0.029–1.875 (data not shown).

### 3.2. ET-1/ETAR Effect on Apoptosis in Breast Cancer Cells

**Figure 3:**
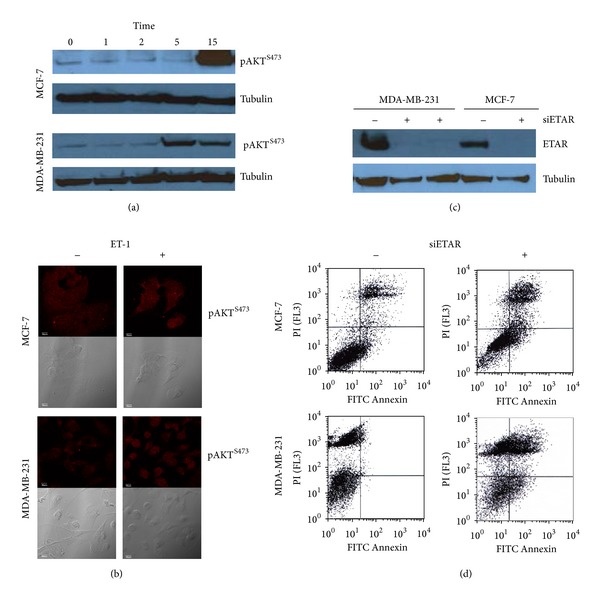
ET-1 stimulatory and ETAR inhibition effects on MCF-7 and MDA-MB-231 cells. Cells were serum deprived for 24 hours and then treated with ET-1 for the indicated times. Resulting cellular lysates were subjected to SDS-PAGE and Western blotting with the indicated antibodies (a). Cells (serum deprived for 24 hours) were treated with ET-1 for 15 minutes, then stained with p-Akt antibody and imaged by confocal microscopy with p-Akt staining (top) or phase contrast (bottom) (b). Silencing of ETAR by siRNA showed decreased ETAR protein by Western blot (c). Apoptosis in both cell lines was determined by flow cytometry using Annexin V and propidium iodide (PI) labeling (d). In the untreated control samples (left upper image for MSF-7 and left lower image for MDA-MB-231), the majority of cells were nonapoptotic (Annexin V−/PI− population). Silencing of ETAR decreased population of nonapoptotic cells and increased population of cells undergoing early apoptosis (Annexin V+/PI−) and late apoptosis (Annexin V+/PI+) as depicted in the images on the right.

We investigated whether ET-1 signaling activates prosurvival pathway as assessed by monitoring phosphorylated Akt in two human breast cancer cell lines: MCF-7 and MDA-MB-231. After stimulation with 10 nM ET-1 for 15 minutes, pAkt was analyzed by semiquantitative Western blot and confocal microscopy. Our results show that ET-1 promotes Akt activation in both breast cancer cell lines (Figures 3(a) and 3(b)). Further experiments were performed to evaluate ET-1/ETAR interactions. Basal ETAR expression in MCF-7 and MDA-MB-231 cells was similar in both cell lines based on semiquantitative Western blot and confocal microscopy results (data not shown). In order to understand the role of ETAR in the survival of breast cancer cell lines, we investigated the fate of breast cancer cells after silencing ETAR. Using RNA interference, we successfully reduced ETAR expression in both cell lines (Figure 3(c)). The determination of apoptosis was done by flow cytometry using dual FITC-labeled annexin V and propidium iodide. Our experiments revealed that siRNA against ETAR increased apoptotic cell population in MCF-7 and MDA-MB-231 cells (Figure 3(d)). These data suggest that the inhibition of ETAR induces apoptosis in both hormone receptor negative and hormone receptor positive breast cancer cells. 

## 4. Discussion 

Our findings indicate that ET-1 expression in tumor and stroma predicts disease-free survival in patients with early breast cancer. We show that patients with ET-1 non-enriched phenotype have an excellent prognosis; however, patients with ET-1-enriched phenotype continue experiencing relapses many years after diagnosis. We propose that ET-1 expression may serve as a prognostic biomarker in the adjuvant breast cancer setting. 

Two-thirds of the cases demonstrated positive ET-1 expression in tumor cells. The finding is in agreement with previous studies, which showed ET-1 positivity in 40–60% of cases [13, 14]. However, in our study we also observed moderate to strong stromal expression of ET-1 in 66% of cases, which is in contrast to the previously reported lack of ET-1 in stromal cells. This discrepancy might be explained by drawbacks of immunostaining techniques such as variation of specimen fixation, choice of antibody, scoring of immunoreactivity and different cut-off values used. We found that patients with high expression of ET-1 in stromal cells were more likely to have high ET-1 expression in tumor cells. Accumulating evidence suggests that cancer stroma is involved in tumor recurrence and therapy resistance. ETs not only stimulate tumor cell growth but also modulate tumor-stroma interactions and further promote tumor progression and metastasis. Several investigators have reported ET-1 expression (by IHC) in epithelial breast cancer cells and trend towards lower DFS in patients with those tumors [7, 14, 15]. In addition, several studies evaluating stromal gene signatures identified ET-1 as one of the genes associated with poor clinical outcome. Finak et al. reported ET-1 as one of the genes linked to angiogenic, hypoxic, tumor-associated macrophage responses and poor breast cancer outcome [16]. Furthermore, Boersma et al. showed upregulation of ET-1 gene in both tumor and stroma of inflammatory breast cancer [17]. 

Unfavorable prognostic factors such as tumor size, tumor grade, and nodal status are all associated with a higher risk of recurrence within the first 5 years of diagnosis [18]. However, among women treated with tamoxifen for 5 years, more than half of all recurrences occur between 6 and 15 years after diagnosis [19]. In our study, the exploratory analysis of ER-positive breast cancer subset did not show the independent prognostic significance of ET-1. Nevertheless, patients with ET-1-enriched tumor phenotype showed a significantly higher risk for recurrence years after diagnosis. Late relapse is well documented in hormone receptor positive breast cancer. The biological mechanisms explaining the difference in risk over time are still unknown, and currently we are unable to predict which patients with ER-positive tumors are at the greatest risk for late recurrence. Extended adjuvant endocrine therapy has shown some additional benefits; however, toxicity, compliance and recurrence remain major issues [20]. Our results support the need for a prospective study to examine whether ET-1-enriched tumor phenotype can identify those hormone receptor positive breast cancer patients with the greatest risk for late recurrence. 

Importantly, our study showed that ET-1 stimulation of breast cancer cells promotes Akt activation. Furthermore, inhibition of ETAR increases apoptosis in breast cancer cells. Those observations suggest that ET-1/ETAR is a functional target in breast cancer. Studies in other tumor types showed that ET-1 acts as an antiapoptotic factor, modulating cell survival pathways through a PI3-K-mediated Akt activation, leading to drug resistance [21, 22]. In addition to apoptosis, several other cell processes, such as invasion, may contribute to cancer therapy resistance [23]. Several investigators demonstrated additive anti-invasive effects of ETAR inhibition in combination with endocrine or HER2 targeted therapies in the preclinical studies [24, 25]. Epithelial mesenchymal transition (EMT) is a state towards a cell invasion and drug resistance [26]. During EMT, epithelial cells acquire mesenchymal features in order to disseminate from the primary tumor but then revert to an epithelial phenotype after reaching the distant site [27]. An activation of PI3 kinase and Akt signaling has been detected in cells undergoing EMT [28]. Furthermore, it has been shown that ET-1/ETAR is implicated in EMT in ovarian cancer, and targeting ETAR in combination with chemotherapy can sensitize tumor to chemotherapeutics by preventing EMT-associated signaling [29, 30]. Nevertheless, the ET-1/ETAR role in EMT progression in breast cancer needs yet to be proven. 

## 5. Conclusions

We found ET-1 expression in tumor and stroma to be an independent prognostic marker for breast cancer recurrence. Patients with ET-1-enriched tumors continue to be at risk for breast cancer relapse many years after diagnosis. Our research findings lay foundation for a validation study to determine whether ET-1 can serve as a biomarker to identify high-risk breast cancer patients at diagnosis for appropriate treatment stratification. We also showed that ET-1/ETAR signaling pathway is functionally associated with cancer related cell processes such as apoptosis. New treatment options are needed for patients with high risk for relapse. For example, extended endocrine therapy for hormone receptor positive tumors or ET-1/ETAR targeted therapy may be further explored in patients with ET-1-enriched tumor phenotype. 

## Figures and Tables

**Table 1:** Demographics, clinicopathologic, and treatment characteristics.

	All patients *N* = 91	Patients without recurrence *N* = 60	Patients with recurrence *N* = 31	*P*-value
	No. (%)	No. (%)	No. (%)	
Age				
Mean (SD)	54.79 (11.52)	55.93 (14.90)	52.58 (13.18)	0.19
Median	55	56	53	
Histology				0.95*
IDC	76 (83.51)	50 (83.33)	26 (83.87)	
ILC	13 (14.28)	8 (13.33)	5 (16.13)	
Other	2 (2.2)	2 (3.33)	0 (0.00)	
Grade				0.56*
Grade 1	18 (19.78)	14 (23.33)	4 (12.90)	
Grade 2	29 (31.87)	19 (31.76)	10 (32.26)	
Grade 3	31 (34.06)	18 (30.00)	13 (48.15)	
Unknown	13 (14.29)	9 (15.00)	4 (12.90)	
Tumor stage				0.42*
T1	52 (57.14)	37 (61.66)	15 (48.39)	
T2	28 (30.76)	16 (26.66)	12 (38.71)	
T3-4	10 (10.98)	6 (10.00)	4 (12.90)	
Unknown	1 (0.11)	1 (1.66)	0 (0.00)	
Nodal Stage				**0.02∗**
N0	56 (61.53)	40 (66.66)	16 (51.61)	
N1	19 (20.87)	14 (23.33)	5 (16.13)	
N2-3	15 (16.48)	5 (8.33)	10 (32.26)	
Unknown	1 (0.11)	1 (1.66)	0 (0.00)	
AJCC Stage				**0.02**
I	42 (46.15)	32 (53.33)	10 (32.26)	
II	31 (34.06)	21 (35.00)	10 (32.26)	
III	18 (19.78)	7 (11.66)	11 (35.48)	
Hormone receptor status				0.09*
ER and PR negative	24 (26.37)	12 (20.00)	12 (38.71)	
ER and/or PR positive	65 (71.42)	46 (76.66)	19 (61.29)	
Unknown	2 (2.19)	2 (3.33)	0 (0.00)	
HER2/neu status				0.62*
Negative	73 (80.22)	50 (83.33)	23 (74.19)	
Positive	13 (14.28)	8 (13.33)	5 (16.13)	
Unknown	5 (5.49)	2 (3.33)	3 (9.68)	
Adjuvant chemotherapy				0.85
Yes	62 (68.1)	40 (66.66)	22 (70.96)	
No	29 (31.9)	20 (33.33)	9 (29.03)	
Adjuvant endocrine therapy				**<0.01**
Yes	63 (69.2)	47 (78.33)	16 (51.61)	
No	28 (30.8)	13 (21.66)	15 (48.38)	
Adjuvant radiotherapy				0.3
Yes	73 (80.2)	50 (83.33)	23 (74.19)	
No	18 (19.8)	10 (16.66)	8 (25.81)	

SD: standard deviation; IDC: invasive ductal carcinoma; ILC: invasive lobular carcinoma.

*Excluding “unknown” or “other” category.

**Table 2:** Multivariable Cox proportional hazard model for DFS: adjusted hazard ratios (HRs) according to age, and nodal stage, ER and HER2 status.

Variable	Adjusted HR	95% CI	*P*-value
Age (per each year)	0.99	0.95–1.03	0.68
Nodal stage			<0.01
0 versus 2-3	0.19	0.06–0.56	
1 versus 2-3	0.18	0.04–0.93	
ER negative versus positive	4.05	1.53–10.72	<0.01
HER2 negative versus positive	0.67	0.24–2.08	0.49
ET-1 non-enriched versus ET-1 enriched	0.16	0.03–0.77	0.02
